# A Comparative Study of the Ease of Radial Artery Cannulation Using the Palpation Method in Patients Undergoing Surgery Under General Anaesthesia: Pre-induction Versus Post-induction

**DOI:** 10.7759/cureus.79115

**Published:** 2025-02-16

**Authors:** Rashmi Dubey, Vikramjit Singh, Monica Khetarpal, Subrata K Singha, Keerty Sharma, Praveen Neema

**Affiliations:** 1 Anaesthesiology, All India Institute of Medical Sciences, Raipur, Raipur, IND; 2 Cardiac Anaesthesiology, Amrita Institute of Medical Sciences, Kochi, IND

**Keywords:** arterial cannulation, general anaesthesia, postinduction, preinduction, radial artery cannulation

## Abstract

Introduction

Radial artery cannulation is performed for continuous blood pressure monitoring and blood gas measurements in the operating room, critical care unit, and postoperative period. Various factors determine the artery used for cannulation, including accessibility, ease of placement, collateral flow, risk of infection, and embolic phenomena. Most anaesthesiologists consider anaesthesia induction the most likely timing of hemodynamic instability. These hemodynamic changes may affect the blood vessels in terms of their lumen and pulsation, thus posing a challenge to the anaesthesiologist for successful arterial cannulation. In the present study, we aim to assess whether these hemodynamic changes, consequent to the induction of general anaesthesia, significantly affect the success rate of radial artery cannulation compared to the success rate if performed before the induction of general anaesthesia using the palpation method.

Materials and methods

A total of 60 eligible and consenting ASA-PS I & II (American Society of Anaesthesiologists Physical Status I & II) patients undergoing elective surgery (which requires invasive blood pressure monitoring) under general anaesthesia were prospectively randomized into two groups, Pre-I (Pre-induction) and Post-I (Post-induction), using a random selection method. In the Pre-I group, radial artery cannulation was performed before induction of anaesthesia, while in the Post-I group, radial artery cannulation was performed after induction of anaesthesia. The data, including the success rate of arterial cannulation, the number of attempts required to cannulate the radial artery successfully, and the time taken to successfully cannulate the radial artery, were obtained and analysed.

Results

Demographic variables such as mean age, weight, height, BMI (body mass index), and the ASA-PS were comparable across the groups, and the differences were not statistically significant. In the Pre-I group, of 30 patients, 28 were in ASA class I, and two were in ASA class II; the respective numbers in the Post-I group were 27 and 3. Of all 60 patients, the radial artery was successfully cannulated in 54 patients - 26 in the Post-I group and 28 in the Pre-I group. The number of attempts for cannulation was one attempt in 23 patients and two attempts in three patients in the Post-I group; the respective numbers for the Pre-I group were 26 and 2. The mean time for cannulation was 14.38 seconds in the Post-I group and 12.67 seconds in the Pre-I group. The complications (hematoma at the site of cannulation) related to radial cannulation were noted in three patients - two in the Post-I group and one in the Pre-I group.

Conclusion

Radial artery cannulation can be performed with comparable ease, before and after the induction of anaesthesia, in patients belonging to ASA-PS I & II.

## Introduction

Arterial catheterization is a common procedure performed in intensive care units, emergency rooms, and operating rooms for continuous blood pressure monitoring and blood gas measurements. Clinicians consider intra-arterial cannulation with continuous blood pressure transduction the most accurate method for measuring and monitoring blood pressure [1]. Indications for placing a radial arterial catheter include the need for real-time, dynamic monitoring of blood pressure in critically ill patients, including those experiencing conditions such as shock, hypertensive emergencies, or stroke, as well as those receiving titratable vasoactive medications. Additionally, it is beneficial for patients undergoing complex surgical procedures, such as neurosurgery, supratentorial surgery, posterior fossa surgery, aneurysm or arteriovenous (AV) malformation surgeries, cardiovascular surgeries such as coronary artery bypass grafting (CABG) or valvular replacement surgeries, surgeries expecting major fluid shifts, and complex gastro surgery, like Whipple's procedure, etc. The catheter also facilitates frequent blood draws, including arterial blood gases (ABGs), for patients on ventilation. Furthermore, when used in conjunction with other technologies, such as pulse pressure variation (PPV), it aids in monitoring cardiac function. Arterial waveform-derived functional hemodynamic parameters like PPV, systolic pressure variation (SPV), and pulse contour analysis-derived parameters - stroke volume variation (SVV) obtained during intra-arterial pressure monitoring - can also be used to predict the physiologic response to fluid resuscitation [2,3]. Various factors determine the artery used for cannulation, including accessibility, ease of placement, collateral flow, risk of infection, and embolic phenomena. Although radial, femoral, dorsalis pedis, brachial, ulnar, and axillary arteries are all accessible sites for cannulation, radial artery cannulation is considered the standard technique for hemodynamic monitoring by many anaesthesiologists [4].

Anaesthesia induction is indeed a critical phase in the perioperative period, and it is often considered the most likely time for hemodynamic instability to occur [5]. Different drugs, such as propofol, thiopentone, or sevoflurane, are used to induce anaesthesia. The effect of these induction agents on hemodynamic parameters, like systolic blood pressure (SBP), mean arterial pressure (MAP), systemic vascular resistance, stroke volume, or cardiac volume, is well established. These induction agents significantly change these hemodynamic parameters, such as decreased MAP, systemic vascular resistance, and increased vasodilatation. These hemodynamic changes may affect the blood vessels in terms of their lumen and pulsation, thus posing a challenge to the anaesthesiologist for successful arterial cannulation [6]. It is expected that these hemodynamic changes are much more pronounced in patients with cardiovascular disease or shock and may lead to hemodynamic collapse. There is enough evidence to show that even brief periods of intraoperative hypotension have ill consequences in such patients and can lead to significant morbidity or mortality [7,8]. With invasive arterial blood pressure monitoring, such episodes can be detected and treated promptly, which may improve the outcome. In such cases, peripheral pulses frequently become impalpable after induction of anaesthesia, and it would seem counterintuitive to insert an arterial line during the post-induction period. So, a common practice is performing arterial cannulation before anaesthesia induction. However, there are no clear guidelines or practice advisories on whether to cannulate the artery before or after the induction of anaesthesia. A thorough literature search has also yet to reveal any study that has assessed the impact of these hemodynamic changes on the success rate of radial artery cannulation. 

In this study, we aimed to evaluate whether the hemodynamic changes that occurred following the induction of general anaesthesia significantly impacted the success rate of radial artery cannulation. We compared the success rates of radial artery cannulation, performed before and after the induction of general anaesthesia, using the palpation method.

We hypothesize that the hemodynamic changes following anaesthesia induction can affect the ease of radial artery cannulation, making it more difficult compared to the pre-induction state.

## Materials and methods

After IEC approval (approval no. AIIMSRPR/IEC/2021/724), this prospective, randomized, and comparative study was conducted in the operating room of the institute. The CTRI registration number is CTRI/2021/08/035624.

The study was designed with meticulous attention to detail, ensuring that all variables were controlled for, and the results were as accurate as possible.

This study aimed to compare the ease of radial artery cannulation using the palpation method before and after the induction of general anaesthesia in patients undergoing elective surgery that required invasive arterial monitoring. The objective was to evaluate any differences in the difficulty of cannulation during these two time periods. The primary outcome of the study was to determine the success/failure rate of radial artery cannulation during the pre-induction and post-induction periods. The secondary outcomes were to calculate the number of attempts required to successfully cannulate the radial artery and to compare the time taken for successful radial artery cannulation between the pre-induction and post-induction periods.

The study included patients aged 18 to 65 years of any gender with ASA-PS I & II (American Society of Anaesthesiologists Physical Status I & II) who underwent elective surgery that required invasive arterial monitoring and radial artery cannulation under general anaesthesia. The study focused on patients undergoing head and neck surgeries, specifically those with carcinoma of the buccal mucosa, cheek, or tongue, who required neck dissection. Patients with supratentorial tumours were also included in the study population. Invasive arterial monitoring was essential for optimal anaesthesia management during these procedures. Patients who refused to give consent had no pulse at the site of arterial cannulation, failed the modified Allen’s test, had Raynaud's syndrome or Buerger's disease, coagulopathies, hypertension, infection at the site of arterial cannulation, second or third-degree burns, artificial vascular grafts, or arterial cannulation site within the proposed surgical field, were excluded from the study.

The sample size was calculated based on the assumption that there would be a minimum of 20% difference in the success rate of radial artery cannulation before and after induction of general anaesthesia, using the palpation method. This assumption was based on the observation that there is typically a 20%-30% decrease in blood pressure following the induction of anaesthesia [9]. The sample size was estimated using the following formula:

\[ N = \frac{\left( Z_{\alpha/2} \sqrt{2p(1-p)} + Z_{1-\beta} \sqrt{p_1(1-p_1)p_2(1-p_2)} \right)^2}{(p_1 - p_2)^2} \]

Here p_1_(0.6) and p_2_(0.8) are success rates, assuming a 20% difference so p_1_ - p_2_ = 0.2. Z_α/2_ = 1.96 at a 95% confidence interval, and Z_β_ = 0.84 at 80% power.

After substituting the values into the formula, the estimated total sample size was 28 for each group. To account for potential dropouts, 30 patients were enrolled in each group.

The study included a total of 60 participants, with 30 individuals in each group: the Pre-I group (Pre-induction group) and the Post-I group (Post-induction group). Participants were randomly assigned to one of the two groups using a simple random sampling technique known as the random selection method. In the Pre-I group, arterial cannulation was performed before the induction period, while in the Post-I group, it was conducted after induction. This randomization process ensures that the groups are comparable, allowing any observed differences to be attributed to the timing of arterial cannulation.

The study variables included the number of attempts for arterial cannulation, criteria for successful/failed arterial cannulation, and the time for successful arterial cannulation. Failed arterial cannulation was defined as three or more attempts at the same site, a change of site for arterial cannulation, or the use of ultrasound for cannulation. The time for successful arterial cannulation was measured as the interval between skin puncture and successful artery cannulation, confirmed by an arterial waveform on the monitor. If the waveform did not correspond to the arterial trace, the cannulation site was changed and considered a cannulation failure.

Data collection procedure

**Figure 1:**
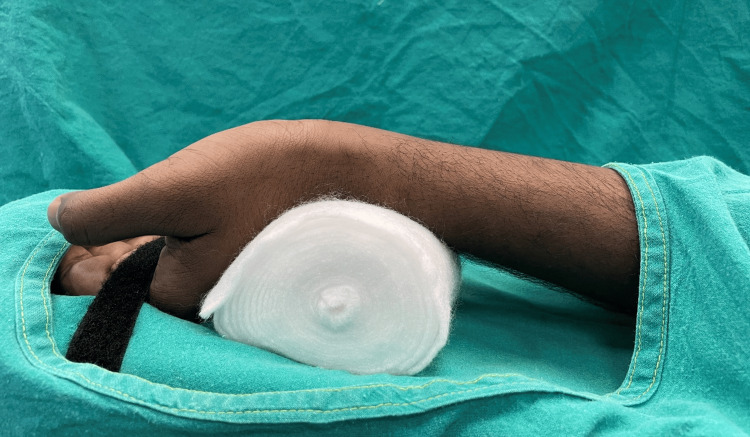
Position of wrist for radial artery cannulation

Following a successful pre-operative evaluation, eligible participants provided consent and were briefed about the study. They were then randomly assigned to either the Pre-I or Post-I group using the random selection method of simple random sampling. A single anaesthesiologist performed the arterial cannulations on all the participants enrolled in the study. Modified Allen's test was performed before radial arterial cannulation to ensure the presence of ulnar-dominant blood flow to the hand. Baseline hemodynamic parameters, heart rate (HR), SBP, and diastolic blood pressure (DBP) were recorded before performing cannulation. The patient's arm was placed over an arm board, and the wrist was hyperextended over a gauge pad placed underneath and held in position by tape, as shown in Figure 1.

**Figure 2:**
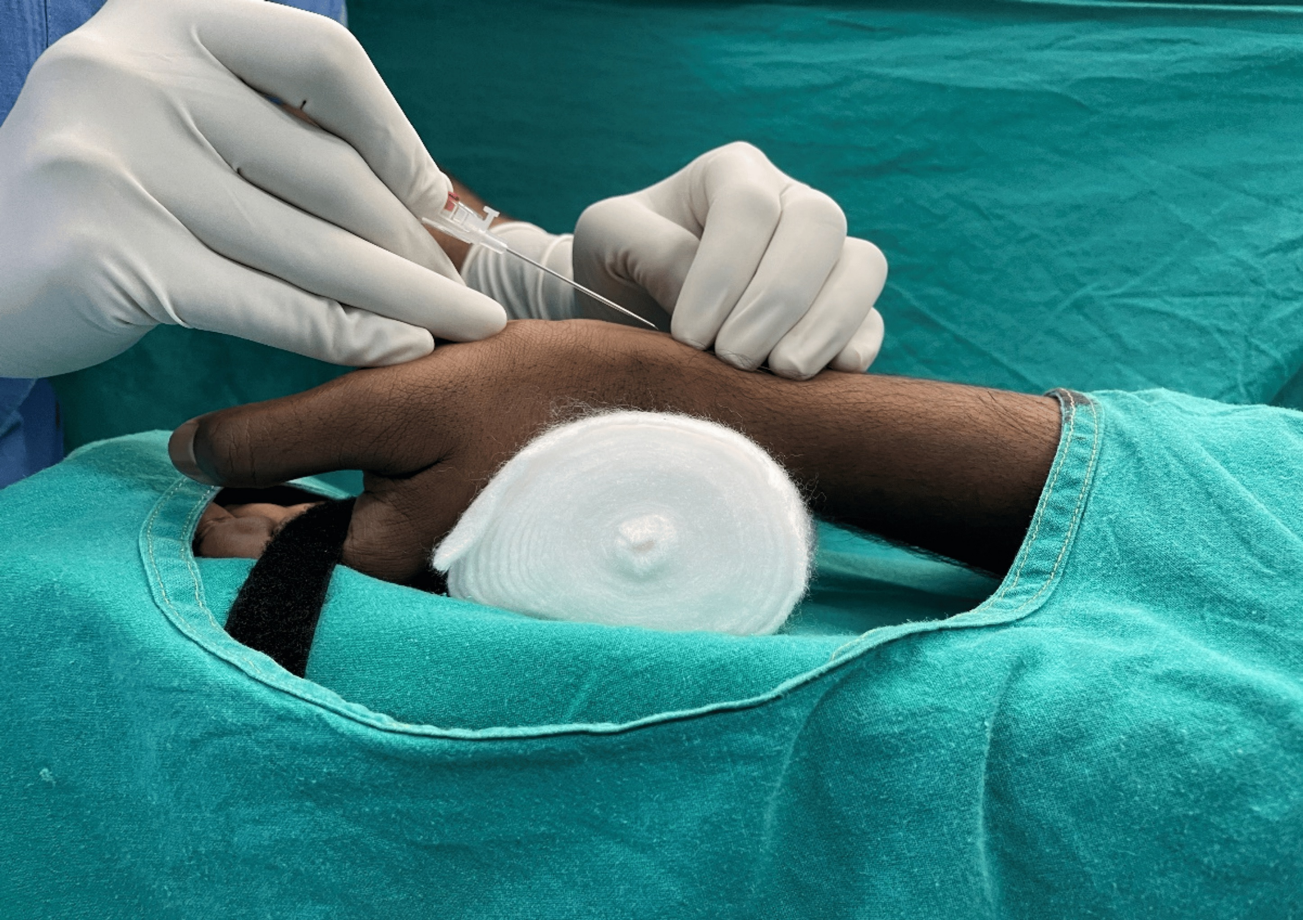
Palpation method of radial artery cannulation

The exposed area was disinfected with iodinated and alcohol-based scrub, and a sizeable sterile drape was placed to maintain an aseptic environment. In all participants, at the arterial cannulation site, an adequate amount of local anaesthetic, lignocaine 2%, was injected with a U-40 insulin syringe. In participants enrolled in the Post-I group, anaesthesia was induced in all the patients with Inj. propofol (dose 1-2.5 mg/kg), Inj. fentanyl (2 µg/kg), and Inj. vecuronium (0.1 mg/kg); isoflurane was used to maintain the depth of anaesthesia while participants were being ventilated with oxygen. Radial artery cannulation was then performed before laryngoscopy and endotracheal intubation to avoid the effect of associated hypertension on study parameters. The artery was palpated using the first and second fingers, and the maximal pulsation point was chosen as the puncture site, typically 1-1.5 cm cephalad from the junction of the arm and the hand. A BD™ Arterial Cannula (20 gauge; BD™, Franklin Lakes, NJ, USA) with a flow switch was used in all participants to cannulate the radial artery. The cannula at the skin puncture site was placed at 45 degrees and gradually advanced; once blood was visible in the hub of the cannula, the cannula was lowered and gently advanced into the artery while holding the needle perfectly still (Figure 2). The catheter was then connected to the pressure tubing and flushed with heparinized saline, and the arterial traces were assessed for the arterial waveform. The catheter was secured using Tegaderm® (3M, St. Paul, MN, USA), a sterile dressing. After successfully inserting the arterial catheter, special care was exercised to ensure that the site, flushing device, and infusion system were contamination-free.

The number of attempts to successfully cannulate the radial artery was counted and recorded. The time for successful arterial cannulation, as defined earlier, was also recorded. The timer was started at the beginning of the skin puncture and stopped when the artery was successfully cannulated. Complications related to arterial cannulation, including hematoma formation and bleeding, were also recorded. The arterial cannula was removed when it was no longer required, or when any sign of circulatory compromise was noted. After removing the catheter, haemostasis was achieved with compression over the arterial puncture site using a gauze swab secured with adhesive plaster tape.

Statistical analysis

Data were analysed using IBM SPSS Statistics for Windows, Version 26 (Released 2020; IBM Corp., Armonk, NY, USA), and GraphPad Prism, Version 5 (GraphPad Software, San Diego, CA, USA) for statistical analysis. Data were summarized as the mean and standard deviation for numerical variables, and frequency and percentages for categorical variables. Two-sample t-tests for a difference in mean involved independent or unpaired samples. Unpaired proportions were compared using the appropriate Chi-square or Fisher's exact test. Once a t-value was determined, a p-value was found using a table of values from Student's t-distribution, and a p-value ≤0.05 was considered statistically significant.

## Results

The demographic variables such as mean age, weight, height, BMI (body mass index), and ASA-PS were similar across all groups, and the differences were not statistically significant (Table 1).

**Table 1:** Distribution of demographic profile (mean age, height, weight, and BMI) between the Post-I and Pre-I groups

Parameter	Group	Mean	SD	Minimum	Maximum	Median	p-value
Age (years)	Post-I	42.86	13.83	17.00	65.00	44.00	0.10
	Pre-I	48.13	11.06	18.00	64.00	48.00	
Height (cm)	Post-I	164.93	7.12	152.00	176.00	164.50	0.74
	Pre-I	165.56	8.06	152.00	179.00	165.00	
Weight (Kg)	Post-I	67.23	8.10	50.00	85.00	68.50	0.34
	Pre-I	69.13	7.43	57.00	85.00	68.00	
BMI (Kg/m^2^)	Post-I	24.77	3.10	18.60	30.50	24.75	0.49
	Pre-I	25.36	3.51	19.90	35.00	25.25	

The baseline vital parameters, including HR, blood pressure (both SBP and DBP), MAP, and peripheral capillary oxygen saturation (SpO₂), were also not statistically significant (Table 2).

**Table 2:** Baseline mean heart rate (bpm), SBP (mmHg), and DBP (mmHg) between the Post-I and Pre-I groups SBP, systolic blood pressure; DBP, diastolic blood pressure

Parameter	Group	Mean	SD	p-value
Heart rate (bpm)	Post-I	95.64	10.34	>0.05
	Pre-I	94.55	10.83	
SBP (mmHg)	Post-I	130.35	8.96	>0.05
	Pre-I	129.36	9.15	
DBP (mmHg)	Post-I	77.63	9.64	>0.05
	Pre-I	76.46	9.37	

Vital parameters, including HR, blood pressure (both SBP and DBP), and MAP just before arterial cannulation, were noted and were statistically significant (Table 3). There was a significant fall in blood pressure (SBP and DBP) and HR after induction.

**Table 3:** Distribution of mean heart rate (bpm), SBP (mmHg), and DBP (mmHg) between the Post-I and Pre-I groups (just before arterial cannulation) SBP, systolic blood pressure; DBP, diastolic blood pressure

Parameter	Group	Mean	SD	Minimum	Maximum	Median	p-value
Heart rate (bpm)	Post-I	70.16	11.34	54.00	91.00	69.00	<0.0001
	Pre-I	82.53	9.83	58.00	97.00	84.50	
SBP (mmHg)	Post-I	99.23	8.96	80.00	115.00	96.00	<0.0001
	Pre-I	127.36	9.95	100.00	142.00	124.00	
DBP (mmHg)	Post-I	58.63	9.64	41.00	80.00	60.00	<0.0001
	Pre-I	76.46	8.37	60.00	90.00	77.50	

Success rate

In the Post-I group, four (13.3%) failures were noted, and arterial cannulation was successful in 26 (86.7%) patients. In the Pre-I group, two (6.7%) failures were noted, and arterial cannulation was successful in 28 (93.3%) patients. The comparison of the success rate of radial artery cannulation between the two groups was not statistically significant, with a p-value of 0.38 (p > 0.05) (Table 4).

**Table 4:** Distribution of success rate between the Post-I and Pre-I groups (shows number of patients and percentage in the bracket) Chi-square value: 0.74; p-value: 0.38

Success rate	Post-I	Pre-I	Total
Fail	4 (13.3333%)	2 (6.667%)	6 (10%)
Success	26 (86.667%)	28 (93.333%)	54 (90%)
Total	30 (100%)	30 (100%)	60 (100%)

Number of attempts

In the Post-I group, 23 (88.5%) patients had arterial cannulation successful on the first attempt, and in three (11.5%) patients, it required two attempts. In the Pre-I group, 26 (92.9%) patients had arterial cannulation successful on the first attempt, and in two (7.1%) patients, it required two attempts. The comparison of the number of attempts required for arterial cannulation between the two groups was not statistically significant, with a p-value of 0.57 (p > 0.05) (Table 5).

**Table 5:** Distribution of the number of attempts between Post-I and Pre-I groups Chi-square value: 0.31; p-value: 0.57

Number of attempts	Post-I	Pre-I	Total
1	23	26	49
2	3	2	5
Total	26	28	54

Mean number of attempts

In the Post-I group, the mean number of attempts (mean ± SD) taken for arterial cannulation was 1.11 ± 0.32. In the Pre-I group, the mean number of attempts (mean ± SD) taken for arterial cannulation was 1.07 ± 0.26. The difference in the mean number of attempts taken for arterial cannulation between both groups was not statistically significant, with a p-value of 0.58 (p > 0.05) (Table 6).

**Table 6:** Distribution of mean number of attempts between Post-I and Pre-I groups

	Group	Mean	SD	Minimum	Maximum	Median	p-value
Mean number of attempts	Post-I	1.11	0.32	1.00	2.00	1.00	0.58
	Pre-I	1.07	0.26	1.00	2.00	1.00	

Time for successful cannulation

In the Post-I group, the mean time for successful cannulation (mean ± SD) of the radial artery was 14.38 ± 4.90 seconds. In the Pre-I group, the mean time for successful cannulation (mean ± SD) of the radial artery was 12.67 ± 3.28 seconds. The difference in mean time for successful cannulation between both groups was not statistically significant, with a p-value of 0.13 (p > 0.05) (Table 7).

**Table 7:** Distribution of mean time for successful cannulation between Post-I and Pre-I groups

	Group	Mean	SD	Minimum	Maximum	Median	p-value
Time for successful cannulation (seconds)	Post-I	14.38	4.90	7.00	25.00	15.00	0.13
	Pre-I	12.67	3.28	6.00	20.00	12.00	

## Discussion

Although it is not known when the first radial artery cannulation was performed, the first recorded cannulation of an arterial blood vessel was conducted in 1714 by the English Reverend Stephen Hales, and the first description of arterial cannulation in a human occurred in 1856 while measuring the blood pressure in the femoral artery [10]. Peterson et al. described the continuous recording of arterial blood pressure during the perioperative period by inserting a plastic catheter into the brachial artery through a metal needle [11].

A Swedish radiologist, Seldinger, described the widely practised catheter-over-wire technique in 1953 [12]. Improvements to catheter design and pressure transducing systems led to increased arterial line use beginning in the 1960s. By 1990, about 8 million arterial catheterizations were performed annually in the United States, most of which were placed in the radial artery [13].

The radial artery is the preferred site of cannulation owing to its accessibility, ease of cannulation by a short catheter, and high physiologic adaptability of the hand's dense arterial network [14-20]. While the optimal methods to evaluate the adequacy of collateral blood flow to the hand are controversial, clinically, Allen's test is most often used to evaluate baseline hand circulation, first described in 1929 by Dr. Allen [21], and later modified by Wright [22] in 1950 to assess flow in a single hand. Ruengsakulrach et al. compared the modified Allen's test with Doppler ultrasonography of the thumb artery in 71 patients and found the modified Allen's test to have a sensitivity of 100% and specificity of 97% [23]. The primary argument against the routine use of the modified Allen's test is the lack of evidence that it can predict hand ischemia after radial artery cannulation. Slogoff et al., after evaluating 411 cardiovascular surgical patients, reported that 3.9% of patients had a recovery time of 15 seconds, and without ischemic complications, radial artery cannulation was performed in these patients [24].

In our study, of all the 60 patients, the radial artery was successfully cannulated in 26 patients in the Post-I group and 28 patients in the Pre-I group. The number of attempts for cannulation was one attempt in 23 and two attempts in three patients in the Post-I group; the respective numbers for the Pre-I group were 26 and 2. The mean time for cannulation was 14.38 seconds in the Post-I group and 12.67 seconds in the Pre-I group. The present study has been planned to determine whether general anaesthesia induction can interfere with the ease of radial artery cannulation. As described above, there was no significant difference in the success rate, attempts needed to cannulate the radial artery, and the time needed to cannulate the radial artery. The data indicate that the study's results do not support the belief that radial artery cannulation is easier if cannulated before the induction of anaesthesia. The belief that radial artery cannulation will be difficult after the induction of anaesthesia is because hemodynamic stability deteriorates. Consistent with the general belief and the reported literature, the mean systolic and mean diastolic arterial pressures decreased significantly in the Post-I group as compared to the Pre-I group (SBP 127.36 ± 9.95 vs. 99.23 ± 8.96 mmHg; DBP 76.46 ± 8.37 vs. 58.63 ± 9.64 mmHg). If mean SBP remains >99 mmHg, the success rate of radial artery cannulation is not affected. As the patients with compromised hemodynamic status were not included in our study, it is inappropriate to comment on the effect of induction of anaesthesia on the success rate of radial artery cannulation in patients with hemodynamic compromise, and it is not possible to negate that induction of anaesthesia in patients with hemodynamic compromise would pose additional difficulty for radial artery cannulation.

It may be noteworthy that the mean BMI of patients in our study was 24.77 ± 3.10 kg/m² in the Post-I group and 25.36 ± 3.51 kg/m² in the Pre-I group, implying that most of the patients fell into the category of Normal or Healthy Weight to Overweight. The number of patients falling into the obesity category was small, with one patient in the Post-I group having a BMI of 30 kg/m² and one with a BMI of 35 kg/m² in the Pre-I group. The palpation of the artery may become challenging in obese individuals and can affect the success rate of arterial cannulation. From the results of our study, it is safe to consider that, in individuals with normal or healthy weights, the success rate of radial artery cannulation is not affected. However, as the number of obese patients was small in our study, we cannot comment on the effect of BMI >30 kg/m² on the success rate of radial artery cannulation.

Our study included patients belonging to ASA-PS I & II only (out of a total of 60 patients, 55 were in ASA-PS I and the remaining five were in ASA-PS II); these patients are normal, healthy individuals and are expected to show marginal hemodynamic changes on induction of anaesthesia, as observed in our study. The SBP in the Pre-I group vs. the Post-I group was 127.36 ± 9.95 vs. 99.23 ± 8.96 mmHg. However, patients belonging to ASA-PS III and above have co-morbid systemic diseases and are expected to show significant hemodynamic changes on induction of anaesthesia. In such patients, it can be challenging to cannulate the radial artery after induction of anaesthesia. Because of ethical concerns, patients of ASA-PS III and above were not included in the study.

Limitations

The results of this study do not apply to patients belonging to ASA-PS III and beyond, or those having a BMI of more than 30 kg/m². A separate study is required to assess the ease of radial artery cannulation in hemodynamically unstable, obese patients with a BMI >30 kg/m², hypovolemic patients, and patients in shock.

## Conclusions

There is no statistically significant difference between the success rate, number of attempts, and time taken for successful radial artery cannulation using the palpation method when performed before and after the induction of anaesthesia. We concluded that radial artery cannulation could be performed with comparable ease, before or after the induction of anaesthesia, in patients belonging to ASA-PS I & II.
